# A new type of Taylor series expansion

**DOI:** 10.1186/s13660-018-1709-8

**Published:** 2018-05-15

**Authors:** Mohammad Masjed-Jamei, Zahra Moalemi, Iván Area, Juan J. Nieto

**Affiliations:** 1 Department of Mathematics, K.N. Toosi University of Technology, Tehran, Iran; 2 Departamento de Matemática Aplicada II, E.E. Aeronáutica e do Espazo, Universidade de Vigo, Vigo, Spain; 3 Instituto de Matemáticas, Universidade de Santiago de Compostela, Santiago de Compostela, Spain

**Keywords:** 41A58, Generalized Taylor expansion, Integration by parts, Integral remainder, Error bound, Jacobi and Laguerre polynomials

## Abstract

We present a variant of the classical integration by parts to introduce a new type of Taylor series expansion and to present some closed forms for integrals involving Jacobi and Laguerre polynomials, which cannot be directly obtained by usual symbolic computation programs, i.e., only some very specific values can be computed by the mentioned programs. An error analysis is given in the sequel for the introduced expansion.

## Introduction

Let $\lbrace x_{j}\rbrace_{j=0}^{n}\in [a,b] $ and $\lbrace f_{j}\rbrace_{j=0}^{n} $, which may be samples of a function, say $f(x) $, be given. The main aim of an interpolation problem is to find an appropriate model to approximate $f(x) $ at any arbitrary point of $[a,b] $ other than $x_{j} $. In other words, if $\Psi(x;a_{0},\ldots,a_{n}) $ is a family of functions of a single variable *x* with $n+1 $ free parameters $\lbrace a_{j}\rbrace_{j=0}^{n} $, then the interpolation problem for Ψ consists of determining $\lbrace a_{j}\rbrace_{j=0}^{n} $ so that, for $n+1 $ given real or complex pairs of distinct numbers $\lbrace (x_{j},f_{j}) \rbrace_{j=0}^{n} $, we have

$$ \Psi(x_{j};a_{0},\ldots,a_{n})=f_{j}. $$

For a polynomial type interpolation problem, various classical methods, such as Lagrange, Newton, and Hermite interpolations, are used. Lagrange’s interpolation as a classical method for approximating a continuous function $f:[a,b]\to\mathbb{R} $ at $n+1$ distinct nodes $a\le x_{0}<\cdots<x_{n}\le b$ is applied in several branches of numerical analysis and approximation theory. It is expressed in the form [1, pp. 39–40]

$$ {\mathrm{P}}_{n}(f;x)=\sum_{j=0}^{n} f(x_{j})\ell_{j}^{(n)}(x) $$

for

$$ \ell_{j}^{(n)}(x)=\frac{w_{n}(x)}{(x-x_{j})w'_{n}(x_{j})}, $$

where $w_{n}(x)=\prod_{j=0}^{n}(x-x_{j})$ is the node polynomial and $\ell_{j}^{(n)}(x) $ are the Lagrange polynomials.

Then ${\mathrm{P}}_{n}(f;x) $ is a unique element in the space of all polynomials of degree at most *n*, say $\mathcal{P}_{n}$, which solves the interpolation problem

$$ {\mathrm{P}}_{n}(f;x_{j})=f(x_{j}),\quad j=0,1,2,\ldots,n. $$

For non-polynomial type interpolation problems, an interpolating function of the form

$$ \psi_{n}(x)=\sum_{j=0}^{n} a_{j}u_{j}(x) $$

is usually considered [2], where $\lbrace u_{j}(x) \rbrace_{j=0}^{n} $ is a set of linearly independent real-valued continuous functions on $[a,b] $ and $\lbrace a_{j}\rbrace_{j=0}^{n} $ are determined by the initial conditions

$$\psi_{n}(x_{j})=f(x_{j}), \quad j=0,1,\ldots,n. $$

The function $\psi_{n}(x) $ exists and is unique in the space of $\text{span}\lbrace u_{j} \rbrace_{j=0}^{n} $ for all $f\in C[a,b] $ if and only if the matrix $\lbrace u_{j}(x_{k}) \rbrace_{j,k=0}^{n} $ is nonsingular.

The general case of an interpolation problem was proposed by Davis [3] containing all the above-mentioned cases. It is indeed concerned with reconstructing functions on a basis of certain functional information, which are linear in many cases. Let Π be a linear space of dimension $n+1 $ and $L_{0},L_{1},\ldots, L_{n} $ be $n+1 $ given linear functionals defined on Π, which are independent in $\Pi^{*} $ (the algebraic conjugate space of Π). For a given set of values $w_{0},w_{1},\ldots , w_{n} $, we can find an element $f\in \Pi $ such that

$$ L_{j}(f)=w_{j},\quad j=0,1,\ldots, n. $$

Hence, one can construct new interpolation formulae using linear operators [4]. For instance, considering $\Pi=\mathcal{P}_{n} $ and linear independent functionals as

$$L_{j}(f)=f^{(j)}(x_{0}),\quad\text{for } j=0,1,\ldots,n , $$

leads to Taylor’s interpolation problem.

Davis also mentioned that the expansion of a function based on a series of predetermined (basis) functions can be interpreted as an interpolation problem with an infinite number of conditions. See also [5] in this regard.

The problem of the representation of an arbitrary function by means of linear combinations of prescribed functions has received a lot of attention in approximation theory. It is well known that a special case of this problem directly leads to the Taylor series expansion where the prescribed functions are monomial bases [6].

The main aim of this paper is to introduce a new type of Taylor series expansion through a variant of the classical integration by parts. In the next section, we present the general form of this expansion and consider some interesting cases of it leading to new closed forms for integrals involving Jacobi and Laguerre polynomials. Also, an error analysis is given in Sect. 3 for the introduced expansion.

## A new type of Taylor series expansion

Let *F* and *G* be two smooth enough functions such that repeated differentiation and repeated integration by parts are allowed for them. The rule of integration by parts [7] allows one to perform successive integrations on the integrals of the form $\int F(t) G(t) \,dt$ without tedious algebraic computations.

By the general rule

$$\int u \,dv=uv- \int v \,du, $$

one obtains

1 $$\begin{aligned} \int_{a}^{b} F(t) G(t) \,dt =& \bigl(F(t) G_{1}(t)-F'(t) G_{2}(t) +\cdots + (-1)^{n-1}F^{(n-1)}(t) G_{n}(t) \bigr) \big\vert _{a}^{b} \\ &{}+ (-1)^{n} \int_{a}^{b}F^{(n)}(t) G_{n}(t) \,dt \\ =& \sum_{k=0}^{n-1}(-1)^{k} \bigl(F^{(k)}(t) G_{k+1}(t) \bigr)\big\vert _{a}^{b} + (-1)^{n} \int_{a}^{b}F^{(n)}(t) G_{n}(t) \,dt, \end{aligned}$$

where $G_{n} $ denotes the *n*th antiderivative of *G*.

Formula (1) provides a straightforward proof for Taylor’s theorem with an integral remainder term, according to the following result.

### Theorem 2.1

*Let* $f\in C^{n+1}[a,b] $ *and* $x_{0}\in [a,b] $. *Then*, *for all* $a\leq x \leq b $, *we have*

2 $$ f(x)=\sum_{k=0}^{n} \frac{1}{k!}(x-x_{0})^{k} f^{(k)}(x_{0}) + \frac{1}{n!} \int_{x_{0}}^{x}f^{(n+1)}(t) (x-t)^{n} \,dt. $$

### Proof

For a classical proof using different arguments, see, e.g., [3]. However, if in (1) one chooses $F(t)=\frac{1}{n!}(x-t)^{n} $, $G(t)=f^{(n+1)}(t) $ and then calculates

$$\frac{1}{n!} \int_{a}^{x} (x-t)^{n} f^{(n+1)}(t) \,dt, $$

formula (2) is obtained. □

For a given function *f*, assume in (1) that $G(t)=f^{(n+1)}(t) $ and $F(t)=p_{n}(x-t) $, where $p_{n}(x)=\sum_{k=0}^{n}c_{k}x^{k} $ is an arbitrary polynomial of degree *n*. So, we have

$$\begin{aligned} & \int_{a}^{x}p_{n}(x-t) f^{(n+1)}(t) \,dt \\ &\quad = \sum_{k=0}^{n-1}(-1)^{k} \biggl( \frac{{\mathrm{d}}^{k}}{{\mathrm{d}}t^{k}}p_{n}(x-t)_{\vert_{t=x}} f^{(n-k)}(x)- \frac{{\mathrm{d}}^{k}}{{\mathrm{d}}t^{k}}p_{n}(x-t)_{\vert_{t=a}} f^{(n-k)}(a) \biggr) \\ &\qquad{} +(-1)^{n} \int_{a}^{x}\frac{{\mathrm{d}}^{n}}{{\mathrm{d}}t^{n}}p_{n}(x-t) f'(t) \,dt \\ &\quad =\sum_{k=0}^{n-1}(-1)^{k} \biggl((-1)^{k}k! c_{k}f^{(n-k)}(x)- \frac{{\mathrm{d}}^{k}}{{\mathrm{d}}t^{k}}p_{n}(x-t)_{\vert_{t=a}} f^{(n-k)}(a) \biggr) \\ &\qquad{} +(-1)^{n} \int_{a}^{x}(-1)^{n}n! c_{n}f'(t) \,dt \\ &\quad =\sum_{k=0}^{n} \biggl( k! c_{k}f^{(n-k)}(x)-(-1)^{k}\frac{{\mathrm{d}}^{k}}{{\mathrm{d}}t^{k}}p_{n}(x-t)_{\vert_{t=a}} f^{(n-k)}(a) \biggr) \\ &\quad =\sum_{k=0}^{n} \bigl( k! c_{k}f^{(n-k)}(x)-p_{n}^{(k)}(x-a) f^{(n-k)}(a) \bigr) , \end{aligned}$$

which is equivalent to

3 $$ \sum_{k=0}^{n}k! c_{k} f^{(n-k)}(x)=\sum_{k=0}^{n}p_{n}^{(k)}(x-a)f^{(n-k)}(a)+ \int_{a}^{x}p_{n}(x-t)f^{(n+1)}(t) \,dt, $$

and can be written as

$$ f(x)=\frac{1}{n! c_{n}} \Biggl( \sum_{k=0}^{n}p_{n}^{(k)}(x-a)f^{(n-k)}(a)- \sum_{k=0}^{n-1}k! c_{k} f^{(n-k)}(x)+ \int_{a}^{x}p_{n}(x-t)f^{(n+1)}(t) \,dt \Biggr). $$

### Remark 1

If $p_{n}(x-t)=\frac{1}{n!}(x-t)^{n} $, then $c_{j}=0 $ for every $j=0,1,\ldots,n-1 $ and $c_{n}=\frac{1}{n!} $. In this case, (3) is reduced to

$$ f(x)=\sum_{k=0}^{n}\frac{(x-a)^{n-k}}{(n-k)!}f^{(n-k)}(a)+ \frac{1}{n!} \int_{a}^{x}(x-t)^{n}f^{(n+1)}(t) \,dt, $$

which is the same as formula (2).

Now, let us consider some particular examples of the main formula (3). We would like to notice here that the closed forms for the integrals involving Jacobi and Laguerre polynomials in the following examples are new in the literature (see, e.g., [8, 9]) to the best of our knowledge, and they can be computed only for specific values of the parameters by using symbolic computation.

### Example 2.2

Let $p_{n}(x)=P_{n}^{(\alpha,\beta)}(x) $ be the Jacobi polynomials [10]. It is known that, for $\alpha,\beta>-1$,

$$ P_{n}^{(\alpha,\beta)}(x)=\sum_{k=0}^{n}C_{k}^{(\alpha,\beta,n)} x^{k}, \quad C_{k}^{(\alpha,\beta,n)}=\sum _{j=k}^{n}(-1)^{j-k} 2^{-j} \begin{pmatrix} n+\alpha+\beta +j\\ j \end{pmatrix} \begin{pmatrix} n+\alpha\\ n-j \end{pmatrix} \begin{pmatrix} j\\ k \end{pmatrix} $$

satisfies the orthogonality relation

$$\int_{-1}^{1}(1-x)^{\alpha}(1+x)^{\beta}P_{m}^{(\alpha,\beta)}(x)P_{n}^{(\alpha,\beta)}(x) \,dx = \biggl( \int_{-1}^{1}(1-x)^{\alpha}(1+x)^{\beta} \bigl(P_{n}^{(\alpha,\beta)}(x) \bigr)^{2} \,dx \biggr) \delta_{m,n}, $$

where

$$\delta_{n,m}= \textstyle\begin{cases} 1, & m=n,\\ 0, & m\neq n. \end{cases} $$

Moreover, they satisfy the important relation

$$ \frac{{\mathrm{d}}^{k}}{{\mathrm{d}}x^{k}}P_{n}^{(\alpha ,\beta)}(x)= \frac{\Gamma(\alpha+\beta+n+1+k)}{2^{k} \Gamma(\alpha+\beta+n+1)}P_{n-k}^{(\alpha+k,\beta+k)}(x),\quad k\leq n. $$

Now, according to (3), we obtain

4 $$\begin{aligned} &\sum_{k=0}^{n}k! C_{k}^{(\alpha , \beta , n)} f^{(n-k)}(x) \\ &\quad =\frac{1}{\Gamma(\alpha+\beta+n+1)}\sum_{k=0}^{n}(x-a)^{k} \sum_{j=0}^{n-k}\frac{1}{2^{j}}f^{(n-j)}(a) \Gamma(\alpha+\beta+n+1+j)C_{k}^{(\alpha+j,\beta+j,n-j)} \\ &\qquad {}+\sum_{k=0}^{n}C_{k}^{(\alpha ,\beta, n)} \int_{a}^{x}(x-t)^{k}f^{(n+1)}(t) \,dt. \end{aligned}$$

For instance, if $f(x)=\frac{1}{1-x} $, then

$$f^{(n-k)}(x)=(n-k)! (1-x)^{-(n-k+1)}, $$

and relation (4) for $a=0 $ and $x<1 $ reads as

$$\begin{aligned} & \int_{0}^{x}P_{n}^{(\alpha,\beta)}(t) (t-x+1)^{-(n+2)} \,dt\\ &\quad =\frac{(1-x)^{-(n+1)}}{n+1}\sum_{k=0}^{n}C_{k}^{(\alpha,\beta,n)} \begin{pmatrix} n\\ k \end{pmatrix}^{-1}(1-x)^{k} \\ &\qquad {}-\frac{1}{(n+1)! \Gamma(n+\alpha+\beta+1)}\sum_{k=0}^{n}x^{k} \sum_{j=0}^{n-k}2^{-j}(n-j)! \Gamma(n+\alpha+\beta+1+j) C_{k}^{(\alpha+j,\beta+j,n-j)}. \end{aligned}$$

Also if for example $f(x)=e^{x} $, then for $a=0 $ we obtain

$$\begin{aligned} e^{x}={}& \frac{1}{\Gamma(\alpha+\beta+n+1)\sum_{k=0}^{n}k! C_{k}^{(\alpha , \beta , n)}}\sum_{k=0}^{n} \sum_{j=0}^{n-k}\frac{1}{2^{j}}\Gamma( \alpha+\beta+n+1+j)C_{k}^{(\alpha+j,\beta+j,n-j)}x^{k} \\ &{} +\frac{1}{\sum_{k=0}^{n}k! C_{k}^{(\alpha , \beta , n)}}\sum_{k=0}^{n}C_{k}^{(\alpha ,\beta, n)} \int_{0}^{x}e^{t}(x-t)^{k} \,dt. \end{aligned}$$

### Remark 2

In Example 2.2, choosing $\alpha=\beta=-\frac{1}{2} $ gives the first kind of Chebyshev polynomials [10] as

$$ T_{n}(x)=\cos (n\arccos x)=\sum_{k=0}^{n}C_{k}^{(n)}x^{n}, $$

with

$$ C_{k}^{(n)}=2^{2n} \begin{pmatrix} 2n\\ n \end{pmatrix}^{-1} \sum_{j=k}^{n}(-1)^{j-k} 2^{-j} \begin{pmatrix} n +j-1\\ j \end{pmatrix} \begin{pmatrix} n-\frac{1}{2}\\ n-j \end{pmatrix} \begin{pmatrix} j\\ k \end{pmatrix}. $$

This means that

$$ T_{n}(x)=\cos (n\arccos x)=2^{2n}{\binom{2n}{n}}^{-1}P_{n}^{(-\frac{1}{2},-\frac{1}{2})}(x). $$

Hence, replacing $p_{n}(x)=T_{n}(x) $ in (3) gives

$$\begin{aligned} &\sum_{k=0}^{n}k! C_{k}^{(n)} f^{(n-k)}(x) \\ &\quad =\sum_{k=0}^{n}\frac{{\mathrm{d}}^{k}}{{\mathrm{d}}x^{k}}\cos \bigl(n\arccos (x-a) \bigr) f^{(n-k)}(a) + \int_{a}^{x}\cos \bigl(n\arccos(x-t) \bigr)f^{(n+1)}(t) \,dt \\ &\quad =\frac{2^{2n}}{(n-1)!}\sum_{k=0}^{n}(x-a)^{k} \sum_{j=0}^{n-k}2^{-j}(n+j-1)! C_{k}^{(j-\frac{1}{2},j-\frac{1}{2},n-j)}f^{(n-j)}(a) \\ & \qquad{}+ \int_{a}^{x}\cos \bigl(n\arccos(x-t) \bigr)f^{(n+1)}(t) \,dt,\quad n\geq1 . \end{aligned}$$

For instance, if $f(x)=e^{x} $, then

$$ \sum_{k=0}^{n}k! C_{k}^{(n)}=e^{a-x} \sum_{k=0}^{n}\frac{{\mathrm{d}}^{k}}{{\mathrm{d}}x^{k}}\cos \bigl(n \arccos (x-a) \bigr)+ \int_{0}^{x-a}e^{-t} \cos (n\arccos t ) \,dt, $$

and for $x=a $ we obtain

$$\begin{aligned} \sum_{k=0}^{n}k! C_{k}^{(n)}&= \sum_{k=0}^{n}\frac{{\mathrm{d}}^{k}}{{\mathrm{d}}x^{k}}\cos \bigl(n \arccos (x) \bigr)_{\vert_{x=0}} \\ &=\frac{2^{2n}}{(n-1)!}\sum_{j=0}^{n}2^{-j}(n+j-1)! C_{0}^{(j-\frac{1}{2},j-\frac{1}{2},n-j)}. \end{aligned}$$

Another special case of Jacobi polynomials is the Legendre polynomials, which are directly derived from the definition of $P_{n}^{(\alpha,\beta)}(x) $ for $\alpha=\beta=0 $ and has the explicit representation [10]

$$ P_{n}(x)=\frac{1}{2^{n}}\sum_{l=0}^{[\frac{n}{2}]}(-1)^{l} \frac{(2n-2l)!}{l! (n-l)! (n-2l)!} x^{n-2l}. $$

Hence, according to (4), we obtain

5 $$\begin{aligned} \sum_{l=0}^{[\frac{n}{2}]}(n-2l)! s_{l}^{(n)}f^{(2l)}(x)={}& \frac{1}{n!}\sum_{k=0}^{n}(x-a)^{k} \sum_{j=0}^{n-k}2^{-j} f^{(n-j)}(a) (n+j)! C_{k}^{(j,j,n-j)} \\ &{} + \int_{a}^{x} P_{n}(x-t) f^{(n+1)}(t) \,dt, \end{aligned}$$

in which

$$ s_{l}^{(n)}=(-1)^{l} \frac{(2n-2l)!}{2^{n} l! (n-l)! (n-2l)!}. $$

For instance, replacing $f(x)=\cos x $ for $a=0 $ in (5) gives

$$\begin{aligned} &\Biggl( \sum_{k=0}^{[\frac{n}{4}]}(n-4k)! s_{2k}^{(n)} - \sum_{k=0}^{[\frac{[\frac{n}{2}]-1}{2}]}(n-2-4k)! s_{2k+1}^{(n)} \Biggr) \cos x \\ &\quad =\frac{1}{n!}\sum_{k=0}^{n}x^{k} \sum_{j=0}^{n-k}2^{-j} (n+j)! \cos \biggl((n-j)\frac{\pi}{2} \biggr) C_{k}^{(j,j,n-j)}\\ &\qquad {}+ \int_{0}^{x} P_{n}(x-t) \cos \biggl(t+(n+1)\frac{\pi}{2} \biggr) \,dt, \end{aligned}$$

generating many new identities for different values of *x*.

### Example 2.3

For $\alpha>-1$, let $p_{n}(x)=L_{n}^{(\alpha)}(x) $ be the Laguerre polynomials [10] given by

$$ L_{n}^{(\alpha)}(x)=\sum_{k=0}^{n}C_{k}^{(\alpha,n)} x^{k}, \quad C_{k}^{(\alpha ,n)}=(-1)^{k} \frac{1}{k!} \begin{pmatrix} n+\alpha\\ n-k \end{pmatrix} . $$

It is known that

$$ \frac{{\mathrm{d}}^{k}}{{\mathrm{d}}x^{k}}L_{n}^{(\alpha)}(x)=(-1)^{k} L_{n-k}^{(\alpha+k)}(x)\quad\text{for any }k\leq n. $$

Hence, according to (3), we have

$$\begin{aligned} \sum_{k=0}^{n}(-1)^{k} \begin{pmatrix} n+\alpha\\ n-k \end{pmatrix} f^{(n-k)}(x)= {}&\sum_{k=0}^{n} \frac{(-1)^{k}}{k!}(x-a)^{k}\sum_{j=0}^{n-k}(-1)^{j} \begin{pmatrix} n+\alpha\\ n-k-j \end{pmatrix} f^{(n-j)}(a) \\ &{}+ \int_{a}^{x}L_{n}^{(\alpha)}(x-t) f^{(n+1)}(t) \,dt. \end{aligned}$$

As a special case, assume that $f(x)=xe^{x} $. Since $\frac{{\mathrm{d}}^{k}}{{\mathrm{d}}x^{k}} (xe^{x})=(x+k)e^{x}$, we get

6 $$\begin{aligned} &e^{x}\sum_{k=0}^{n}(-1)^{k} \binom{n+\alpha}{n-k}(x+n-k) \\ &\quad =e^{a} \sum_{k=0}^{n} \frac{(-1)^{k}}{k!}(x-a)^{k}\sum_{j=0}^{n-k}(-1)^{j} \begin{pmatrix} n+\alpha\\ n-k-j \end{pmatrix} (a+n-j) \\ &\qquad {}+\int_{a}^{x}(t+n+1) e^{t} L_{n}^{(\alpha)}(x-t) \,dt. \end{aligned}$$

For instance, if $x=1 $ and $a=0 $ in (6), then

7 $$\begin{aligned} &\int_{0}^{1}(n+2-t) e^{-t} L_{n}^{(\alpha)}(t) \,dt \\ &\quad =\sum_{k=0}^{n}(-1)^{k} \begin{pmatrix} n+\alpha\\ n-k \end{pmatrix} (n-k+1) -e^{-1}\sum_{k=0}^{n} \frac{(-1)^{k}}{k!}\sum_{j=0}^{n-k}(-1)^{j} \begin{pmatrix} n+\alpha\\ n-k-j \end{pmatrix} (n-j) . \end{aligned}$$

For $\alpha=0$, the right-hand side of (7) can be expressed in terms of hypergeometric series and evaluations of Laguerre polynomials as

$$n _{1}F_{0}(1-n;\ ;1)+ _{1}F_{0}(-n;\ ;1)+ \frac{n^{2} _{1}F_{1}(1-n;2;1)-L_{n-1}^{(0)}(1)}{e (n-1)}. $$

For $\alpha \neq 0$, the right-hand side of (7) can be written as

$$ \frac{ (\alpha n+\alpha +n^{2}+n-1 ) \Gamma (\alpha +n-1)}{\Gamma (\alpha ) \Gamma (n+1)} - e^{-1} \sum_{k=0}^{n} \frac{(-1)^{k}}{k!} \frac{(n (\alpha +n)-k) \Gamma (\alpha +n-1)}{\Gamma (\alpha +k) \Gamma (-k+n+1)}, $$

where the latter sum can be expressed in terms of hypergeometric series as

$$\begin{aligned} &\sum_{k=0}^{n} \frac{(-1)^{k}}{k!} \frac{(n (\alpha +n)-k) \Gamma (\alpha +n-1)}{\Gamma (\alpha +k) \Gamma (-k+n+1)} \\ &\quad = \frac{ _{1}F_{1}(1-n;\alpha +1;1) \Gamma (\alpha +n-1)}{\Gamma (\alpha +1) \Gamma (n)} +\frac{(\alpha +n) \Gamma (\alpha +n-1)}{\Gamma (\alpha ) \Gamma (n)} {}_{1}F_{1}(-n; \alpha ;1) . \end{aligned}$$

For more certain new, interesting, and useful integrals and expansion formulas involving the hypergeometric function and the Laguerre polynomials, see [11].

## Error analysis

It is clear that relation (3) can be considered as an approximation. This means that the expression $\sum_{k=0}^{n}k! c_{k} f^{(n-k)}(x)$ can be approximated by $\sum_{k=0}^{n}p_{n}^{(k)}(x-a)f^{(n-k)}(a) $, which is indeed a polynomial of degree *n*. Hence, the exact remainder [3] of this approximation reads as

$$ E_{n}(x;f)= \int_{a}^{x}p_{n}(x-t)f^{(n+1)}(t) \,dt. $$

Now, if $f\in C^{n+1}[a,b] $, a direct result for the corresponding error term is that

$$\begin{aligned} \bigl\vert E_{n}(x;f) \bigr\vert &= \biggl\vert \int_{a}^{x}p_{n}(x-t)f^{(n+1)}(t) \,dt \biggr\vert \leq \int_{a}^{x} \bigl\vert p_{n}(x-t)f^{(n+1)}(t) \bigr\vert \,dt \\ &\leq M_{n} \int_{a}^{x} \bigl\vert p_{n}(x-t) \bigr\vert \,dt, \end{aligned}$$

where $M_{n}=\max_{a\leq t \leq x}\vert f^{(n+1)}(t)\vert $.

Moreover, if the polynomial $p_{n}(\cdot) $ is nonnegative on $[0,x-a] $, e.g., when the coefficients $\lbrace c_{k}\rbrace_{k=0}^{n} $ are all nonnegative, then we have

$$\int_{a}^{x} \bigl\vert p_{n}(x-t) \bigr\vert \,dt= \int_{0}^{x-a} p_{n}(t) \,dt =\sum _{k=0}^{n}\frac{c_{k}}{k+1}(x-a)^{k+1} , $$

and therefore

$$\bigl\vert E_{n}(x;f) \bigr\vert \leq M_{n} \sum _{k=0}^{n}\frac{c_{k}}{k+1}(x-a)^{k+1} . $$

For instance, let us consider the function $f\in C^{(n+1)}[0,1] $ and choose the polynomial as

$$p_{n}(x)=\sum_{k=0}^{n} \frac{1}{2^{k}} x^{k}. $$

Then we obtain

$$\bigl\vert E_{n}(x;f) \bigr\vert \leq \max_{0\leq t \leq x} \bigl\vert f^{(n+1)}(t) \bigr\vert \sum_{k=0}^{n} \frac{1}{2^{k}(k+1)} x^{k+1}. $$

As another example, consider the polynomial $p_{n}(x)=\sum_{k=0}^{n} \frac{(m)_{k}}{k!} x^{k} $ for $0< m<1 $, where $(m)_{k}=\prod_{j=0}^{k-1}(m+j) $ is the Pochhammer symbol. If $f\in C^{(n+1)}[0,1] $, then for any $x\in [0,1] $ we obtain

$$\bigl\vert E_{n}(x;f) \bigr\vert \leq \max_{0\leq t \leq x} \bigl\vert f^{(n+1)}(t) \bigr\vert \sum_{k=0}^{n} \frac{(m)_{k}}{k! (k+1)} x^{k+1} = \max_{0\leq t \leq x} \bigl\vert f^{(n+1)}(t) \bigr\vert x \sum_{k=0}^{n} \frac{(m)_{k} (1)_{k}}{(2)_{k}} \frac{x^{k}}{k!}. $$

Now if $n\rightarrow\infty $, then we get

8 $$ \bigl\vert E_{n}(x;f) \bigr\vert \leq \max _{0\leq t \leq x} \bigl\vert f^{(n+1)}(t) \bigr\vert x \sum _{k=0}^{\infty}\frac{(m)_{k} (1)_{k}}{(2)_{k}} \frac{x^{k}}{k!} = \max_{0\leq t \leq x} \bigl\vert f^{(n+1)}(t) \bigr\vert x _{2}F_{1}(m,1;2;x), $$

where $_{2}F_{1}(m,1;2;x) $ denotes the Gauss hypergeometric function [12]. For instance, replacing $f(x)=e^{x} $ in (8) yields

$$\bigl\vert E_{n}\bigl(x;e^{x}\bigr) \bigr\vert \leq x e^{x} _{2}F_{1}(m,1;2;x), $$

and the error bound for $x=1 $ can be computed as

$$\bigl\vert E_{n}\bigl(1;e^{x}\bigr) \bigr\vert \leq e _{2}F_{1}(m,1;2;1)=\frac{\Gamma(1-m)}{\Gamma(2-m)} e, $$

where we have used the Gauss formula [13, 14]

$$_{2}F_{1}(a,b;c;1)=\frac{\Gamma(c) \Gamma(c-b-a)}{\Gamma(c-b) \Gamma(c-a)}. $$
